# Transcranial direct current stimulation alters sensorimotor modulation during cognitive representation of movement

**DOI:** 10.3389/fnhum.2022.862013

**Published:** 2022-10-06

**Authors:** Gaia Bonassi, Giovanna Lagravinese, Martina Putzolu, Alessandro Botta, Marco Bove, Elisa Pelosin, Laura Avanzino

**Affiliations:** ^1^S.C. Medicina Fisica e Riabilitazione Ospedaliera, ASL4, Azienda Sanitaria Locale Chiavarese, Chiavari, Italy; ^2^Department of Neuroscience, Rehabilitation, Ophthalmology, Genetics, Maternal and Child Health, University of Genoa, Genoa, Italy; ^3^Ospedale Policlinico San Martino, IRCCS, Genoa, Italy; ^4^Section of Human Physiology, Department of Experimental Medicine, University of Genoa, Genoa, Italy

**Keywords:** motor imagery, sensorimotor modulation, short afferent inhibition, primary motor area, transcranial magnetic stimulation

## Abstract

We recently demonstrated, by means of short latency afferent inhibition (SAI), that before an imagined movement, during the reaction time (RT), SAI decreases only in the movement-related muscle (sensorimotor modulation) and that a correlation exists between sensorimotor modulation and motor imagery (MI) ability. Excitatory anodal transcranial direct current stimulation (a-tDCS) on M1 could enhance the MI outcome; however, mechanisms of action are not completely known. Here, we assessed if a-tDCS on M1 prior to an MI task could affect sensorimotor modulation. Participants imagined abducting the index or little finger in response to an acoustic signal. SAI was evaluated from the first dorsal interosseus after the “go” signal, before the expected electromyographic (EMG) activity. Participants received 20-min 1.5 mA a-tDCS or sham-tDCS on M1 on two different days, in random order. Results showed that a-tDCS on M1 increases the sensorimotor modulation consisting of a weakening of SAI after the Go signal with respect to sham-tDCS, in the movement-related muscle right before the beginning of MI. These results suggest that a-tDCS on M1 further potentiate those circuits responsible for sensorimotor modulation in the RT phase of MI. Increased sensorimotor modulation during MI may be one of the mechanisms involved in MI improvement after a-tDCS over M1.

## Introduction

Motor imagery (MI) is the mental simulation of movement without muscle activity (Jeannerod and Decety, 1995), and shares many aspects with actual movement. Indeed, it is well known that through MI, as in motor execution (ME), it is possible to improve the speed and accuracy of a known action (Gueugneau et al., 2013; Avanzino et al., 2015), but also to learn a complex sequence of movements (Bonassi et al., 2017). In fact, MI activates similar brain areas during ME even if to a lesser extent (Grèzes and Decety, 2001), including the primary motor area (M1).

Motor preparation typically precedes movement and is thought to determine the properties of upcoming movements.

It has been shown that also for motor preparation, MI and ME share similar mechanisms. There is neurophysiological evidence related to the use of sensory information during motor preparation of MI and ME. Indeed, we recently demonstrated that sensorimotor integration is modulated in the preparation phase of both MI and ME (Bonassi et al., 2019). Specifically, we studied sensorimotor integration by means of transcranial magnetic stimulation (TMS) protocol, i.e., the short afferent inhibition (SAI) (Tokimura et al., 2000) before the onset of imagined movements (Bonassi et al., 2019). SAI was reduced in the movement-related muscle before the expected movement onset in a MI task, in a reaction time (RT) task (Bonassi et al., 2019). By means of this experiment, we showed that sensorimotor modulation operates during the cognitive representation of movement (i.e., MI) with selective disinhibition of the cortical representation of the muscle involved in the task. We hypothesized that the selective sensorimotor modulation prior to MI was reflective of sensory gating mechanisms, a prevalent physiological process was important for information filtering in sensorimotor systems (Bonassi et al., 2019). Sensory gating is believed to reflect an active suppression or cancelation of the predicted sensory consequences of an action to make the system more sensitive to unexpected sensations and involves subcortical (thalamus) and cortical (S1) neural structures (Macerollo et al., 2018). Therefore, the selective extraction of relevant sensory input and suppression of irrelevant information allows humans to effectively plan and execute the movement.

We also demonstrated that SAI modulation in the motor preparation phase of MI has behavioral relevance. Indeed, we observed a correlation between the magnitude of sensorimotor modulation and the self-perceived ability to imagine the same movement explored during the experimental paradigm, suggesting that the stronger the somatosensory modulation, the better a subject performed kinesthetic imagery of the movement (Bonassi et al., 2019).

Based on the evidence that transcranial direct current stimulation (tDCS) acts on modifying neuronal excitability, the possibility to use tDCS to boost MI has been explored and results demonstrated that combining anodal (excitatory) tDCS (a-tDCS) on M1 with MI enhances the effect of these two techniques applied alone (Saimpont et al., 2016). Although the exact mechanisms are not entirely understood; tDCS has been shown to alter the resting membrane potential, thereby changing the excitability of the stimulated area (for review see Stagg and Nitsche, 2011).

To date, how a-tDCS could act on M1 for improving MI is still not clear. Following our previous results on sensorimotor modulation, here, we tested the hypothesis that one mechanism of action by which a-tDCS applied over M1 acts for improving MI is through the modulation of the sensorimotor integration process (SAI) in the preparation phase of MI. This hypothesis was driven by the following evidence present in the literature.

First, even if it is still not clear whether SAI results from direct thalamocortical projections to M1 or *via* a relay through the primary somatosensory cortex, SAI is the result of inhibition within M1. The sensory input is supposed to exert its inhibitory effects on the layer V pyramidal neurons through γ-Aminobutyric acid (GABA)-ergic intracortical circuits (Di Lazzaro et al., 2005; Di Lazzaro and Ziemann, 2013), but its magnitude is also modulated by dopaminergic and cholinergic neuromodulatory circuits (Van Der Zee et al., 1996). Thus, by changing the neuronal excitability in M1 by means of a-tDCS could theoretically modulate SAI.

Second, tDCS on M1 can modulate subcortical–cortical networks in the human brain (such as thalamus–cortical interaction) by changing the levels of various neurotransmitters, such as GABA, dopamine, and acetylcholine (Scelzo et al., 2011; Pellicciari et al., 2013). Following this evidence, a-tDCS could either influence subcortical structures implicated in sensory gating or modulate neurotransmitter mechanisms underlying SAI.

## Materials and methods

### Subjects

Twenty right-handed subjects (mean age 22.35 ± 1.95, 6 men and 14 women) were recruited for this study. All subjects were in good health, without any nervous, muscular, orthopedic, or cognitive disorders. Right arm dominance was determined by means of the Edinburgh Handedness Inventory (Oldfield, 1971). The experimental protocol was approved by the ethics committee of the University of Genoa and was carried out in agreement with legal requirements and international norms (World Medical Association, 2013). Informed consent was acquired from all the participants before the experiment.

### Transcranial direct current stimulation

A direct current stimulator (BrainSTIM, E.M.S. s.r.l.) delivered a constant current of 1.5 mA, through two sponge electrodes (surface area of 25 cm^2^) in a saline-soaked solution. To increase cortical excitability of M1, in the active stimulation condition, the anode electrode was placed over the left M1, located using C3 in accordance with the international 10–20 system of measurement, while the cathode was placed over the contralateral supraorbital area (anodal tDCS, a-tDCS) (Nitsche et al., 2008). The stimulation session lasted for 20 min. In the SHAM stimulation condition (s-tDCS), electrodes were placed similarly to the active condition, the current was ramped-up for 20 s until it reached 1.5 mA, then ramped down for 20 s and turned off without the participant's knowledge, so that the participant felt the same sensation of active stimulation. This sham condition has been confirmed to produce no effects on brain excitability (Dissanayaka et al., 2018). The order of the stimulation conditions was randomized and counterbalanced across subjects.

### Electromyographic recording

Electromyography was recorded with silver disc surface electrodes placed on a tendon belly arrangement over the bulk of the right first dorsal interosseus (FDI) and abductor digiti minimi (ADM) muscles. Electromyography signals were amplified and filtered (20 to 1 kHz) with a D360 amplifier (Digitimer). The signals were sampled at 5,000 Hz, digitized with a laboratory interface (Power 1401, Cambridge Electronic Design, Cambridge, UK), and stored on a personal computer for display and later offline data analysis.

### Transcranial magnetic stimulation

Single pulses were delivered using a Magstim 200 stimulator (Magstim, UK) with a monophasic current waveform connected to a figure-of-eight-shaped coil (external diameter of each loop, 9 cm). The coil was placed tangentially to the scalp, with the handle pointing postero-laterally at an approximate angle of 45 held tangentially to the scalp, with a posterior–anterior current direction. The coil was positioned over the hand area of the left M1. An optimal coil position (hot spot) was selected so as to elicit the largest motor evoked potential (MEP) in the right FDI. For obtaining single-pulse MEPs (single), the TMS intensity was adjusted to elicit 1 mV peak-to-peak amplitude (SI1 mV). The SI1 mV was expressed as a percentage of the maximum stimulator output (% MSO).

#### Short latency afferent inhibition protocol

Conditioning electrical stimuli (ES) were delivered over the median nerve at the right wrist through a bipolar electrode (cathode proximal) using a square-wave pulse (duration, 200 μs) set at an intensity just above the threshold for evoking a small twitch in the opponens pollicis muscle (Digitimer D180 high voltage electric stimulator). The ES preceded the test TMS shock. The interstimulus interval between the ES and TMS stimuli was set at 20 ms) (Tokimura et al., 2000). The intensity of the TMS shock was set at SI1 mV. The triggers for electrical stimulation and TMS were generated by the Power 1401 (Cambridge Electronic Design, CED, Cambridge, UK). SAI was tested by collecting ten conditioned trials (COND: ES+ TMS) and 10 unconditioned test trials (TMS only).

### Experiment procedures

**Figure 1:**
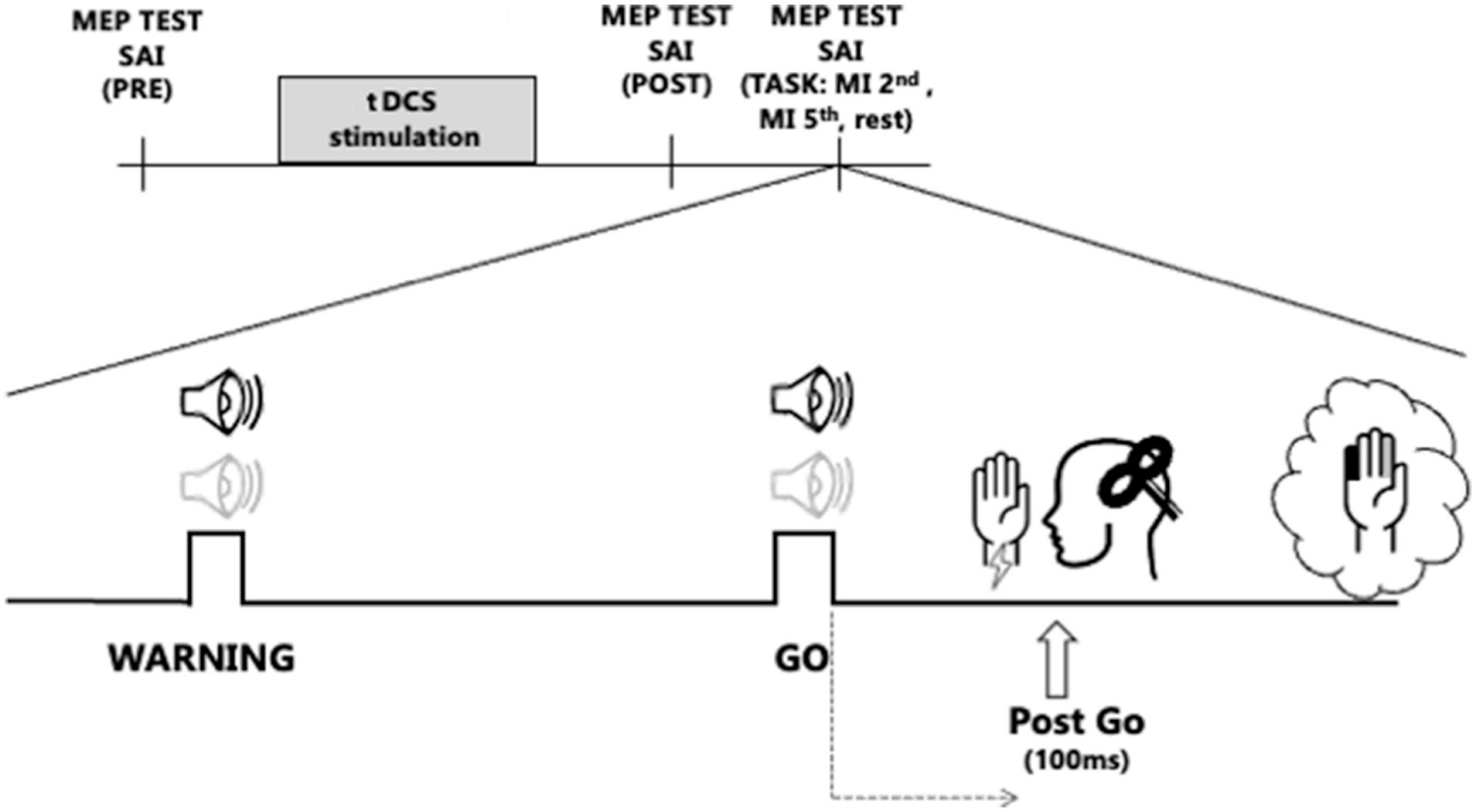
Experimental protocol. The transcranial direct current stimulation (tDCS) stimulation block represents the two stimulation protocols, anodal tDCS (a-tDCS) or sham tDCS (s-tDCS), delivered before the RT task, over M1. Sound icons represent Warning and Go cues. The light gray audio symbol represents the tone for the 2nd finger imagined abduction, while the black stands for the 5th finger abduction. Vertical bar indicates the testing times of short latency afferent inhibition (SAI) from the first dorsal interosseus muscle, before tDCS (SAIpre), after tDCS (SAIpost), and 100 ms after the Go signal (Post-Go) in the following conditions: at rest (Rest) and during imagined movement of the 2nd and the 5th finger (2nd finger MI, 5th finger MI).

The Experimental procedures are shown in Figure 1. A flow chart to illustrate the whole experimental paradigm has also been added as Supplementary material. Subjects were comfortably seated at a desk with their eyes closed. After the determination of the hot spot of the right FDI muscle, the stimulation intensity to elicit single-pulse MEPs of 1 mV was obtained. SAIpre (at baseline, before a-tDCS or s-tDCS) was tested. Then, all subjects received blinded stimulation of tDCS and two tDCS conditions were randomly applied to the subjects in separate sessions. The session interval was 1 week: in the two different sessions we applied a-tDCS or sham-tDCS over left M1. After t-DCS, the single-pulse TMS intensity was readjusted to elicit test MEPs of 1 mV (adjusted single), if needed, and SAI was tested again (SAIpost, immediately after tDCS). Then subjects completed the experimental task, and SAI was tested in different conditions, while subjects were engaged in the task.

The experimental task consisted of an imagined RT task: subjects were asked to kinesthetically imagine of moving a finger as a response to an acoustic cue. Two distinct sounds were associated with different fingers: a high tone for the index finger (2nd) and a low tone for the little finger (5th). Sound cues were produced with a customizable microcontroller board (Arduino Uno). Every trial included two acoustic sounds, a Warning cue, to let participants know which finger they were supposed to imagine moving, and, 2–3 s later, a Go cue after which they had to start imagining the abduction of the selected finger. If the “warning” acoustic cue was a high tone, the required imagined movement, after the Go cue was the index finger abduction (2nd finger). If it was a low tone, the imagined movement was the little finger abduction (5th finger). The experimental task always started with a brief after a brief familiarization session in which participants were trained to mentally perform a sound reaction task of finger abduction, in response to an acoustic cue.

The SAI was tested in the following three conditions during the experimental task: (i) during the RT task, 100 ms after the Go cue while participants were imagining moving the 2nd (SAIpost 2nd finger MI) or the 5th (SAIpost 5th finger MI) finger and (ii) at rest (SAIpostRest), randomly during the experiment, when no movement was asked to participants, to ensure to test SAI in the same attentional status as during the MI task.

Ten conditioned test trials (COND: ES+ TMS) and ten unconditioned test trials (TMS only) were collected for each condition. In total, subjects were instructed to imagine 20 movements with the 2nd finger and 20 movements with the 5th finger. The experiment took around 20 min to complete. The triggers for electrical stimulation and TMS were temporally synchronized with the auditory signals thanks to CED Signal Software.

### Data analysis

The mean MEP amplitudes were calculated from the peak-to-peak amplitudes of 10 trials in each of the SAI recording sessions, separately for conditioned and unconditioned trials.

The amplitude of the conditioned MEPs was expressed as a ratio of the mean unconditioned response (SAI = $\frac{\;COND}{\;TEST}$). To emphasize sensorimotor modulation, the SAI value at the post-Go testing time was further normalized to the SAI at rest (SAI _ratio =_ $\frac{SAI\;post-Go}{SAI\;at\;rest}$) (Bonassi et al., 2019). This normalization shows data as an increase or a decrease of SAI. A value greater than 1 indicates an SAI reduction in relation to rest, whereas a value of less than 1 indicates an increase in SAI in relation to rest (Asmussen et al., 2014; Bonassi et al., 2019).

### Statistical analysis

We checked that all variables were normally distributed (Shapiro–Wilk test) and that sphericity was respected (Mauchly tests). Two-way repeated measures analysis of variance (RM-ANOVA) was used to analyze SI1 mV changes (% MSO) induced by tDCS. The factors for the ANOVA were *Neuromodulation* (a-tDCS and s-tDCS) and *Time* (pre, post).

Data collected at baseline assessment of SAI (SAIpre) was compared to data collected immediately after tDCS (SAIpost) by means of a two-way Repeated Measures ANOVA (RM-ANOVA) with *Neuromodulation* and *Time*) as within-subject factors.

The SAI data were entered into a two-way RM-ANOVA with Neuromodulation and Task (Rest, 2nd finger MI, 5th finger MI) as within-subject factors to assess the impact of neuromodulation on SAI collected during participation in the experimental task. Furthermore, SAI_ratio_ data were entered in a two-way RM-ANOVA with *Neuromodulation* and *Task* (2nd finger MI, 5th finger MI) and as within-subject factors.

Statistical analysis was performed with SPSS 22.0. The *p*-values of 0.05 were considered the threshold for statistical significance. *Post-hoc* analysis of significant interactions was performed by means of *t*-tests applying the Bonferroni correction for multiple comparisons when necessary.

## Results

Statistical analysis on % MSO revealed a significant interaction effect of *Neuromodulation* × *Time* [*F*_(1, 19)_ = 7.54; *p* = 0.013]. *Post-hoc* analyses showed that after a-tDCS, SI1 mV significantly decreased at post (*p* = 0.010) compared with that at pre (pre, 53 ± 4.61%; post 51 ± 4.35%), whereas after the s-tDCS SI1 mV at post (*p* = 0.39) was not different from that at pre (pre, 53.55 ± 5.72%; post 53.1 ± 4.9%).

Statistical analysis on SAI data collected before and after the administration of a-tDCS or s-tDCS revealed no effect for *Neuromodulation* [*F*_(1, 19)_ = 0.42; *p* = 0.52] or *Time* [*F*_(1, 19)_ = 1.99; *p* = 0.17], nor for the interaction *Neuromodulation* × *Time* [*F*_(1, 19)_ = 0.19; *p* = 0.66; Table 1].

**Table 1:** Short latency afferent inhibition (SAI), expressed as a ratio of the amplitude of the conditioned MEPs respect to that of the mean unconditioned response (SAI = $\frac{\text{COND}}{\text{TEST}}$), collected before and after anodal transcranial direct current stimulation (a-tDCS) or sham (s-tDCS) over M1.

**a-tDCS**		**s-tDCS**	
**PRE**	**POST**	**PRE**	**POST**
0.31 ± 0.06	0.32 ± 0.07	0.29 ± 0.09	0.31 ± 0.08

Mean ± SD are reported.

**Figure 2:**
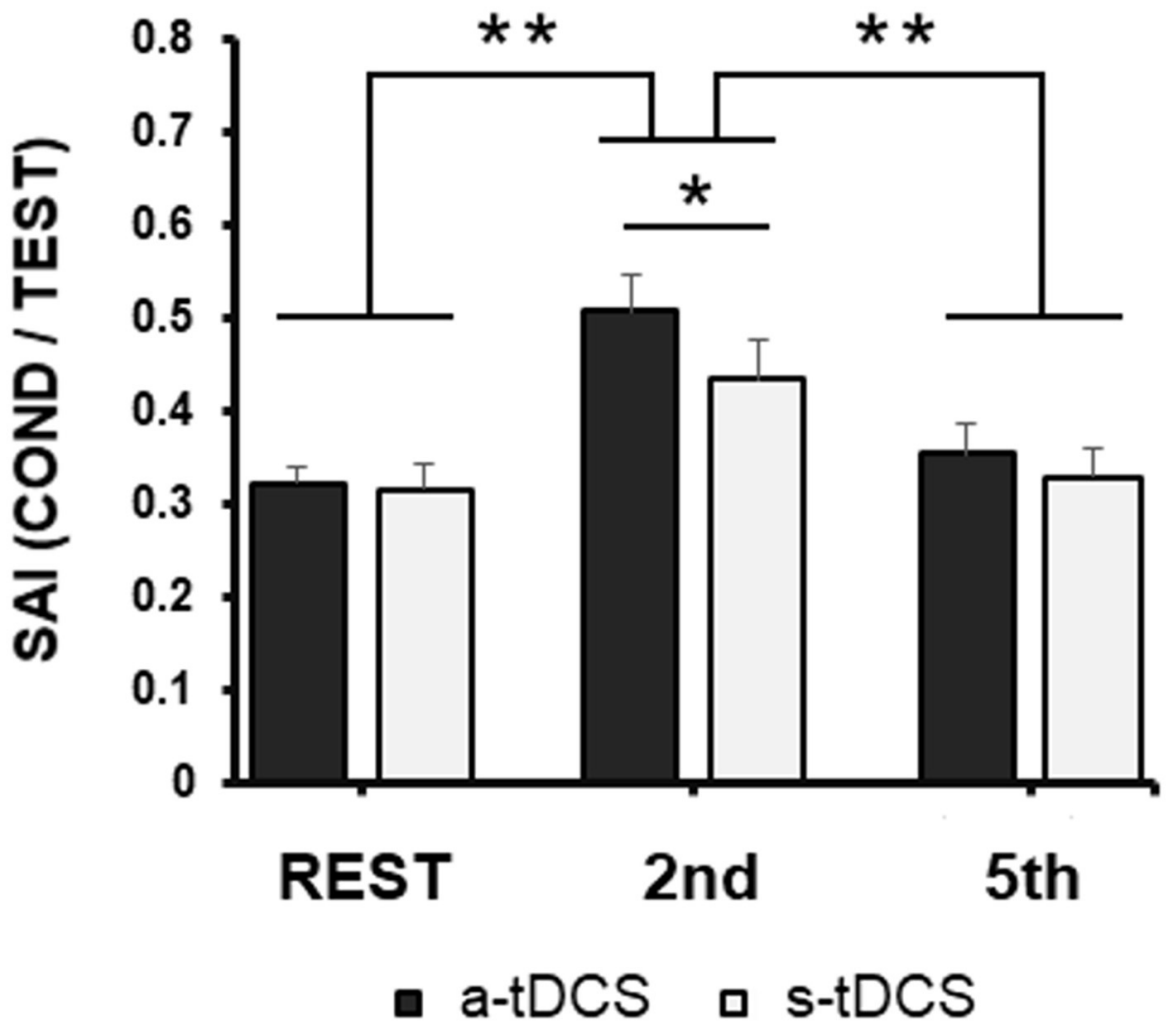
Group averaged data (with standard error of the mean) of SAI modulation tested from FDI before 2nd and 5th fingers movements imagination or at rest during the experimental task, collected after a-tDCS (black columns) or s-tDCS (white columns). Vertical bars indicate SE; asterisks indicate the level of significance (* refers to *p* < 0.05, ** refers to *p* < 0.01).

When we compared SAI recorded during the task in the different conditions (Rest, 2nd finger MI, 5th finger MI; Figure 2) in the two different days (after a-tDCS or s-tDCS, respectively), RM-ANOVA did not highlight any neuromodulation main effect [*F*_(1, 19)_ = 1.95; *p* = 0.17], but showed a significant effect of *Task* [*F*_(2, 38)_ = 31.63; *p* < 0.001] and a significant *Task* × *Neuromodulation* [*F*_(2, 38)_ = 5.27; *p* = 0.009] interaction. *Post-hoc* analysis of the interaction showed the following results.

First, confirming the results of our previous study (Bonassi et al., 2019), sensorimotor modulation occurred in the RT of an MI task, in a very specific way: after both a-tDCS and s-tDCS when SAI was recorded after the Go signal, in the RT of 2nd finger MI, the inhibition resulted lower than when SAI was recorded after the Go signal in the RT of 5th finger MI (*p* < 0.001) or at rest (*p* < 0.001), with no difference between these two latter conditions (*p* = 0.30).

Related to tDCS effect, SAI assessed at rest, but while patients were engaged in the experimental paradigm, did not differ between a-tDCS and s-tDCS sessions (*p* = 0.90). Similarly, when SAI was recorded after the Go signal, in the RT of 5th finger MI, SAI did not differ between a-TDCS and s-tDCS sessions (*p* = 0.31).

We found a significant difference between the a-tDCS and s-tDCS sessions only when SAI was recorded after the Go signal, in the RT of 2nd finger MI (*p* = 0.045): SAI was decreased further when the task was performed after a-tDCS with respect to s-tDCS.

**Figure 3:**
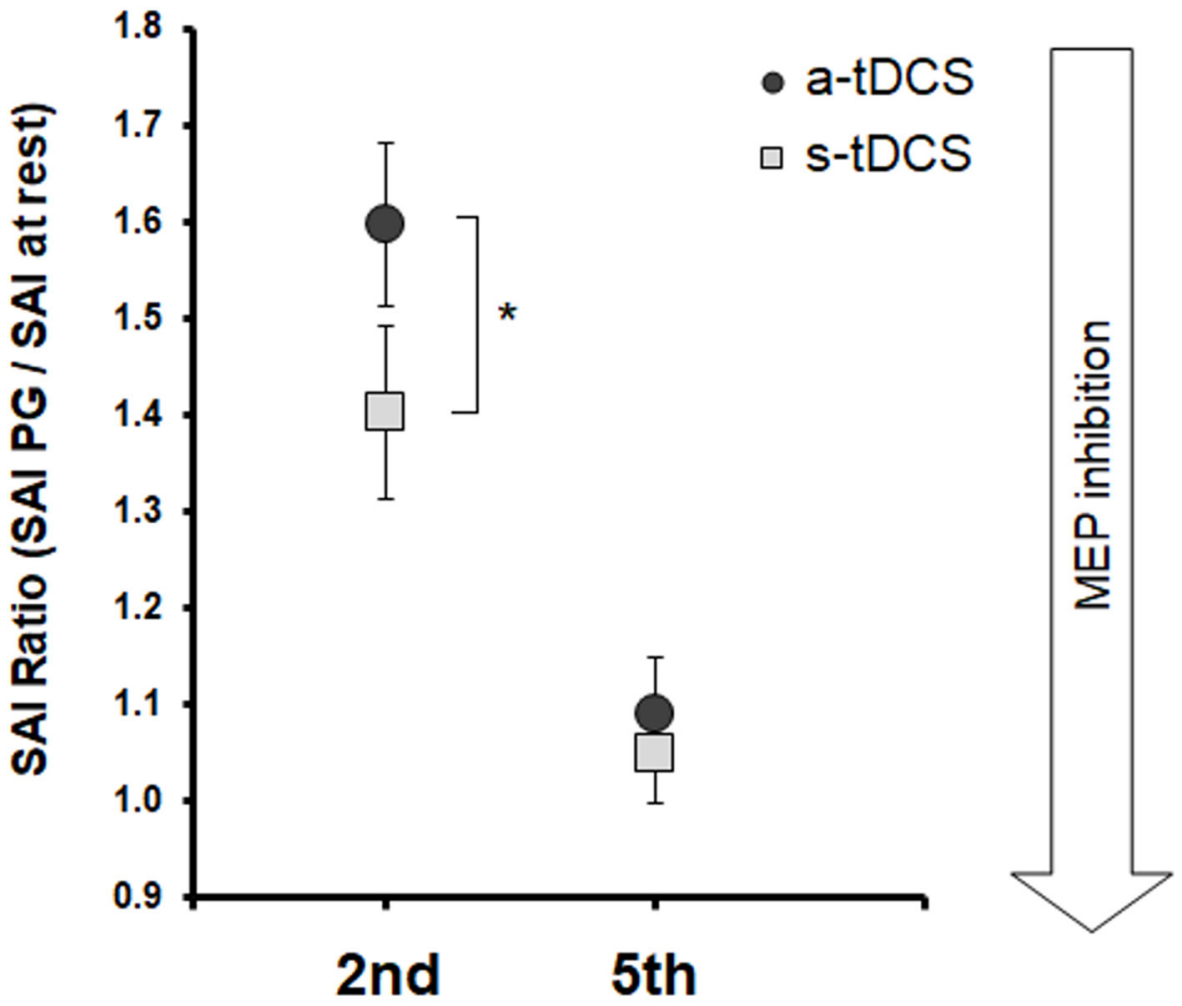
Group averaged data (with standard error of the mean) of SAI Ratio, tested from FDI before 2nd and 5th fingers movement imagination, collected after a-tDCS (dark gray circles), or s-tDCS (light gray squares). Vertical bars indicate SE; asterisks indicate the level of significance (* refers to *p* < 0.05, ** refers to *p* < 0.01).

Statistical analysis on SAI_ratio_ values (Figure 3) showed a significant effect of *Neuromodulation* [*F*_(1, 19)_ = 11.21; *p* = 0.003] and of *Task* [*F*_(1, 19)_ = 28.41; *p* < 0.001] and *Task* x *Neuromodulation* interaction [*F*_(1, 19)_ = 5.87; *p* = 0.025]. *Post-hoc* analysis of the *Task* factors confirmed that SAI decreased (higher values of SAI_ratio_) after both a-tDCS and s-tDCS when the to-be-moved finger was the same to be tested (2nd finger) with respect to when the to-be-moved finger was different to the one tested (5th finger; *p* < 0.001). *Post-hoc* analysis of the *Task* x *Neuromodulation* interaction showed that SAI modulation on FDI was stronger after a-tDCS stimulation with respect to s-tDCS stimulation only in the RT of the imagined 2nd finger movement (2nd finger movement, a-tDCS vs. s-tDCS, *p* < 0.001; 5th finger movement, a-tDCS vs. s-tDCS, *p* = 0.44).

## Discussion

The aim of the study was to explore whether sensorimotor modulation in the preparation phase of a MI task is influenced by a-tDCS on M1. To this aim, we applied a-tDCS over M1, and we tested sensorimotor modulation, during a sound RT task, prior to imagine movement.

First, our results confirmed those of our previous study: there was a sensorimotor modulation, consisting in a decrease of the inhibition of M1 excitability exerted by the peripheral nerve stimulation, in the RT phase of an imagined movement, but only in the case of homotopic stimulation (when SAI was assessed from right FDI during 2nd finger abduction) and not in the case of heterotopic stimulation (when SAI was assessed from right FDI during 5th finger abduction).

Regarding the effect of excitatory tDCS applied over M1, our results showed that a-tDCS on M1 exerted no effect on the excitability of the network involved in SAI when SAI was tested at rest or after the Go signal, in the RT of 5th finger MI. Differently, a-tDCS on M1 exerted an effect on sensorimotor modulation mechanisms occurring in the RT phase of a MI task: the sensorimotor modulation consisting of a weakening of SAI after the Go signal was potentiated after a-tDCS compared to s-tDCS, in the movement-related muscle right before the beginning of MI.

These results suggest that excitatory tDCS on M1 further potentiates those circuits responsible for sensorimotor modulation (decrease of the afferent inhibition exerted by the electrical stimulus over M1 excitability) occurring in the RT phase of the imagined movements, only in the case of homotopic stimulation.

A possible mechanism hypothesized for sensorimotor modulation occurring during imagined movements is the one dealing with a gating mechanism of the sensory inputs coming from the selected muscle. Sensory gating during movement might be due to both bottom-up, centripetal mechanisms, together with top-down, centrifugal mechanisms originating from cortical and subcortical structures (Macerollo et al., 2018). Recordings of sensory gating during mental movement simulation, highlight the role of top-down signals in the sensory gating mechanism (Cheron and Borenstein, 1992). Following this hypothesis, we can speculate that the weakening of SAI during the RT of an imagined movement may be mediated by the influence of a-tDCS on thalamus-sensory network connectivity. Some functional effects of tDCS, likewise long-lasting pain relief in chronic pain patients, have been attributed to a suppression of thalamic sensory pathways following M1 stimulation (Vaseghi et al., 2015). It has been recently demonstrated that a-tDCS on M1 modulates functional connectivity between somatosensory regions of the thalamus and sensory networks (Sankarasubramanian et al., 2017).

Alternatively, increased sensorimotor modulation in the cortical representation of the finger to be moved in the MI task can potentially be the consequence of selective disinhibition (reduced activity of GABAergic interneurons) of the M1 cortical circuits controlling that finger. Recent evidence showed that M1 cell responsiveness increase to TMS during MI could be mediated, at least in part, by a decrease of inhibitory activity within M1 (Grosprêtre et al., 2016; Neige et al., 2020). SAI has been shown to be due to reduction of later I waves, especially I3 (Tokimura et al., 2000; Ni et al., 2011) believed to arise from cortico–cortical interaction (likely the interaction between primary sensory cortex, S1 and M1) (Di Lazzaro et al., 2012). a-tDCS over M1 has been shown to modulate the excitability of S1: the N20-P25 component of the somatosensory evoked potentials recorded from S1 decreased immediately after a-tDCS of M1 (Vaseghi et al., 2015). Suppression of N20-P25 amplitude after M1 stimulation may be explained by activation of the projections from motor to sensory cortex (Lee et al., 2010; Chen et al., 2013). These projections mainly affect areas 1 and 2 of the sensory cortex.

Following this speculation, suppression of S1 activity may in turn have influenced the activity in S1-M1 cortico–cortical interaction involved in sensorimotor modulation, potentiating a cortical disinhibition mechanism engaged in MI. However, we cannot exclude that a-tDCS also directly modulated S1 excitability, since the surface of the electrode placed over M1 was 25 cm^2^. The precise spatial localization of the postulated mechanisms may be assessed in future studies using high-density tDCS. Another drawback of the current study is that here we explored the effect of anodic stimulation on M1, comparing it only to sham stimulation; however, cathodal stimulation should be used to test any potential opposite effects on SAI.

To conclude, whatever is the exact mechanism, here we showed that a-tDCS on M1 acts on selective sensorimotor modulation occurring in the RT of a MI task. This finding may suggest that one of the mechanisms involved in the improvement of MI performance after a-tDCS over M1 is by increasing sensorimotor modulation during MI, since the magnitude of sensorimotor modulation has been associated with MI ability. A better knowledge of the mechanisms of action by which anodal tDCS over the M1 boosts, MI is instrumental to the definition of tailored neuromodulation protocols to enhance motor imagery training in the rehabilitation field.

## Data availability statement

The raw data supporting the conclusions of this article will be made available by the authors, without undue reservation.

## Ethics statement

The experimental protocol was approved by the Ethics Committee of the University of Genoa. The patients/participants provided their written informed consent to participate in this study.

## Author contributions

GB and LA: data analysis, writing, and finalizing manuscript. GB, GL, MP, and AB: data collection and data analysis. GB, LA, MB, and EP: data analysis and drafting the manuscript. All authors contributed to the article and approved the submitted version.

## Funding

This work was supported by the grant from Italian Ministry of Health (Ricerca Corrente) to LA.

## Conflict of interest

The authors declare that the research was conducted in the absence of any commercial or financial relationships that could be construed as a potential conflict of interest.

## Publisher's note

All claims expressed in this article are solely those of the authors and do not necessarily represent those of their affiliated organizations, or those of the publisher, the editors and the reviewers. Any product that may be evaluated in this article, or claim that may be made by its manufacturer, is not guaranteed or endorsed by the publisher.
