# Supplementary figures and images for: Transcranial direct current stimulation alters sensorimotor modulation during cognitive representation of movement
Source: Front Hum Neurosci. 2022 Oct 6;16:862013. doi: 10.3389/fnhum.2022.862013 (PMC9583391; doi:10.3389/fnhum.2022.862013)

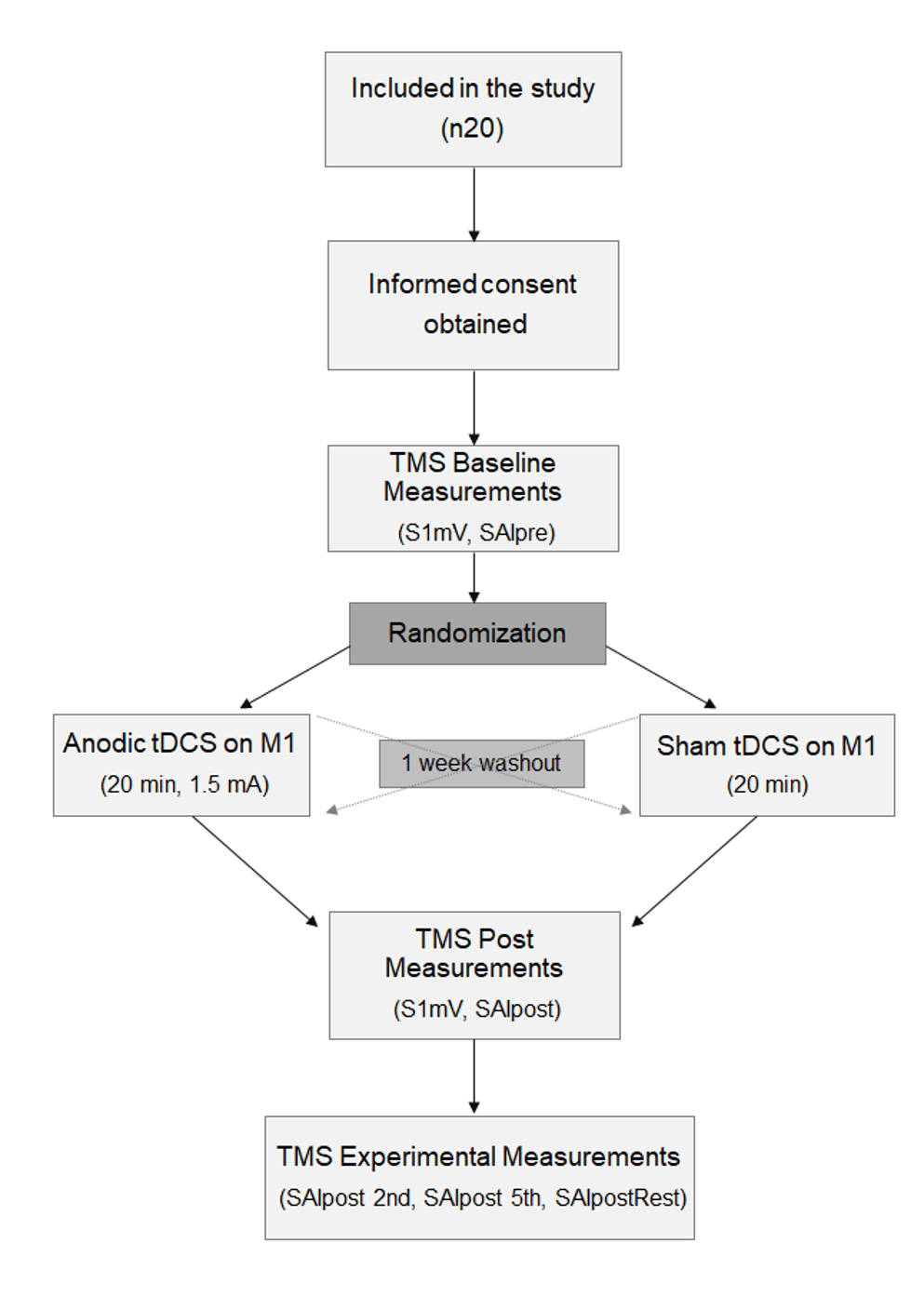

Supplement: Supplementary file 1 [file Image_1.TIFF]
